# Stripped-down DNA repair in a highly reduced parasite

**DOI:** 10.1186/1471-2199-8-24

**Published:** 2007-03-20

**Authors:** Erin E Gill, Naomi M Fast

**Affiliations:** 1 Department of Botany, University of British Columbia, Vancouver, British Columbia, Canada

## Abstract

**Background:**

*Encephalitozoon cuniculi *is a member of a distinctive group of single-celled parasitic eukaryotes called microsporidia, which are closely related to fungi. Some of these organisms, including *E. cuniculi*, also have uniquely small genomes that are within the prokaryotic range. Thus, *E. cuniculi *has undergone a massive genome reduction which has resulted in a loss of genes from diverse biological pathways, including those that act in DNA repair.

DNA repair is essential to any living cell. A loss of these mechanisms invariably results in accumulation of mutations and/or cell death. Six major pathways of DNA repair in eukaryotes include: non-homologous end joining (NHEJ), homologous recombination repair (HRR), mismatch repair (MMR), nucleotide excision repair (NER), base excision repair (BER) and methyltransferase repair. DNA polymerases are also critical players in DNA repair processes.

Given the close relationship between microsporidia and fungi, the repair mechanisms present in *E. cuniculi *were compared to those of the yeast *Saccharomyces cerevisiae *to ascertain how the process of genome reduction has affected the DNA repair pathways.

**Results:**

*E. cuniculi *lacks 16 (plus another 6 potential absences) of the 56 DNA repair genes sought via BLASTP and PSI-BLAST searches. Six of 14 DNA polymerases or polymerase subunits are also absent in *E. cuniculi*. All of these genes are relatively well conserved within eukaryotes. The absence of genes is not distributed equally among the different repair pathways; some pathways lack only one protein, while there is a striking absence of many proteins that are components of both double strand break repair pathways. All specialized repair polymerases are also absent.

**Conclusion:**

Given the large number of DNA repair genes that are absent from the double strand break repair pathways, *E. cuniculi *is a prime candidate for the study of double strand break repair with minimal machinery. Strikingly, all of the double strand break repair genes that have been retained by *E. cuniculi *participate in other biological pathways.

## Background

### DNA repair in eukaryotes

DNA repair processes are vital to all living organisms. Without appropriate mechanisms to remove and replace damaged bases and nucleotides, multiple lesions would accumulate, leading to total genome degradation and loss of vital genetic information. DNA lesions take many forms, including single strand and double strand breaks, in addition to inter- and intra-strand crosslinks and modified bases. Several pathways operate in a concerted manner to minimize information loss each time a DNA lesion occurs. Many of these fundamental pathways have been conserved throughout eukaryotes, and many eukaryotic enzymes have homologues in prokaryotes [1].

**Figure 1:**
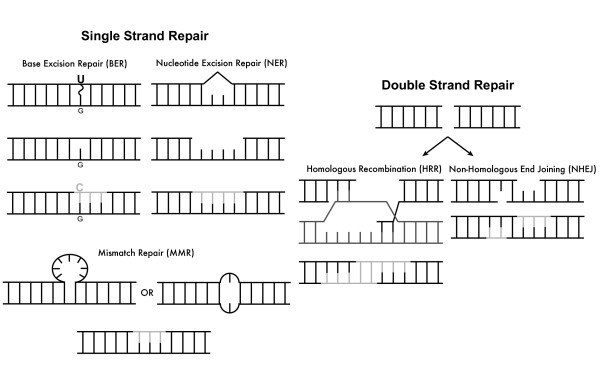
**A comparison of the five major DNA repair pathways**. (See text for explanation.) Newly synthesized DNA is indicated in grey.

Eukaryotic DNA repair can be divided into six primary pathways, all of which are conserved [2]. Some types of DNA lesions (such as double stranded breaks) can be recognized and repaired by more than one pathway. Therefore there is some overlap in function between pathways [2]. Mismatch repair (MMR), base excision repair (BER) and nucleotide excision repair (NER) all operate to repair aberrant bases or nucleotides from one strand of the double helix, using the other strand as a template for new DNA synthesis. In contrast, methyltransferase repair does not require the synthesis of new DNA; anomalous methyl groups are removed without causing any breaks in the double helix. Non-homologous end joining repair (NHEJ) and homologous recombination repair (HRR) are double strand break repair pathways [2]. Double strand breaks are one of the most detrimental forms of DNA lesions, as they can cause genome fragmentation and apoptosis if they are not properly repaired [3]. (See Figure 1 for a comparison of DNA repair processes.)

DNA polymerases are key players in DNA repair, as they are required to fill in gaps created by repair enzymes or incurred from damage [4]. Eukaryotic cells have a wide range of polymerases that are specialized in function; certain polymerases act almost solely in genome replication, others are only active at DNA lesions, and a few have dual roles in repair and replication [4].

For consistency, the names of the genes and proteins involved in these pathways will be referred to using *Saccharomyces cerevisiae *nomenclature.

### Genome reduction in *Encephalitozoon cuniculi*

*Encephalitozoon cuniculi *belongs to a group of obligate intracellular parasites known as microsporidia. These organisms infect a variety of animals including fish, insects and mammals. Various microsporidia have gained the attention of the medical community in the past few decades due to their infection of immuno-compromised humans, such as AIDS and chemotherapy patients [5].

Microsporidian genomes range in size from 2.3 Mbp to 19.5 Mbp. The only completely sequenced microsporidian genome, that of *E. cuniculi*, is a mere 2.9 Mbp in size [6]. The *E. cuniculi *genome is smaller than that of other eukaryotes for several reasons: it has fewer and shorter genes that are separated by tiny intergenic spaces and are only interrupted by a few short introns [6]. Given the degree of genome reduction, the effects are evident in most cellular processes, including DNA repair.

The precise phylogenetic position of microsporidia is not yet known, but a large body of evidence indicates that they are closely related to the fungi [6–10 and others]. Therefore, *E. cuniculi*'s DNA repair systems have been compared primarily to those of another fungus, the yeast *S. cerevisiae*. *S. cerevisiae*'s repair pathways have been well studied at the functional level, making this organism ideal for comparative purposes. In order to gain an accurate perspective of what genes have been lost from *E. cuniculi *during the process of genome reduction (i.e., genes that were present in the common ancestor of microsporidia and fungi), only genes that have homologues in animals were examined.

## Results

### DNA repair inventory

Comparison of DNA repair proteins in *S. cerevisiae *to *E. cuniculi*'s genome and proteome via BLAST and PSI-BLAST searches has revealed that *E. cuniculi *appears to contain a reduced set of proteins in all major repair pathways. Of the 56 repair genes that were sought in *E. cuniculi*, 16 are absent, with another 6 potentially absent. Six out of 14 DNA polymerases or polymerase subunits are absent (See Table 1). Although all repair pathways have been reduced, the loss of genes is not distributed evenly among pathways. Each process has been affected differently by genome reduction. A detailed discussion of the components of each pathway is presented below.

### Base excision repair (BER)

BER is one of the least complex of the DNA repair mechanisms, and involves only a small number of proteins. When a base becomes damaged, it is recognized by a DNA glycosylase that is specific for the particular base and/or the type of damage (methylation, oxidation, etc.). *S. cerevisiae *contains four types of glycosylases, although far more have been found in other organisms (animals, bacteria, etc.) [11]. Glycosylases cleave the glycosylic bond between the base and the deoxyribose to remove the damaged base, at which point a non-specific apurinic/apyrimidinic (AP) endonuclease (Apn1 or Apn2) removes the remaining deoxyribose phosphate to create a gap [12]. In short patch BER (which replaces a single nucleotide), the gap is filled by DNA polymerase β. In long patch BER (which replaces two or more nucleotides), the DNA polymerases β, or δ and ε in concert with proliferating cell nuclear antigen (PCNA) synthesize several nucleotides which displace the original DNA strand. Rad27 then removes the displaced DNA. The ligase Cdc9 (or DNA ligase III and Xrcc1 in other eukaryotes) is used to seal the nick [13,14].

The Rad1-Rad10 and Mus81-Mms4 endonucleases are also believed to play minor roles in BER by processing the 3' ends of the DNA once an incision has been made into the sugar-phosphate backbone [12].

*E. cuniculi*'s BER pathway appears to be nearly complete, but lacks DNA polymerase β, the Cdc9 DNA ligase (but possesses Xrcc1, the cofactor of the ligase used in this process in some eukaryotes) and part of a 3' endonuclease, Mms4. (See Table 1) Deletion of either polymerase β or Mms4 is not a lethal mutation in yeast, however *S. cerevisiae *cannot survive in the absence of Cdc9 [15]. Another ligase is likely utilized for BER in *E. cuniculi*, as sharing non-specialized enzymes between pathways is not uncommon in *S. cerevisiae *(see discussion), and there is no reason to believe that this is not the case in *E. cuniculi*.

### Nucleotide excision repair (NER)

NER is used primarily to remove bulky lesions from DNA, such as inter- and intra-strand crosslinks. NER is a more complex process than BER, and utilizes a large number of proteins that are evolutionarily conserved among eukaryotes. NER is comprised of two subpathways, global genome repair (GGR) and transcription-coupled repair (TCR). As is suggested by their names, the subpathways act on different types of DNA: DNA that is not transcribed (or the non-transcribed strands of expressed genes), and actively transcribed DNA, respectively. In both GGR and TCR, DNA damage recognition is the first step to occur, followed by DNA unwinding. Next, incisions are made on either side of the aberrant base(s), and a total of 25–30 nucleotides on either side are removed as a single strand. The gap is then filled by DNA polymerase and sealed by DNA ligase. Recruitment of five multi-protein complexes, nucleotide excision repair factors (NEFs) 1 through 4 and the replication protein A (RPA) complex, is believed to take place in a stepwise manner to complete this process. The RPA complex is composed of Rpa1 and Rpa2 and recognizes damaged DNA.

In GGR, the first protein complex to arrive at the damaged site is NEF4, which recognizes damage and is composed of the proteins Rad7 and Rad16. Rad7 binds the NEF2 complex (Rad4/Rad23), recruiting it to the damaged site and increasing DNA binding efficiency. The presence of NEF4 is not strictly required for the recruitment of NEF2 to the DNA lesion, but facilitates the process. The above proteins do not act in the other sub-pathway; in TCR, initiation of repair takes place when an RNA polymerase stalls. Two proteins involved specifically in TCR, Rad26 and Rad28, also participate in the beginning of this process [16].

In both GGR and TCR, NEF1 and NEF3 are the next components to be recruited, and are held at the damage site by NEF2. NEF1 is composed of Rad1, Rad10 and Rad14, while NEF3 is composed of Rad2 and transcription elongation factor IIH (TFIIH). TFIIH contains the Rad3, Rad25, SSL1, TFB1, TFB2 and TFB3 proteins, and provides the single strand DNA helicases required for repair proteins to access the damaged site. Rad1 and Rad10 form a heterodimer that acts as a single strand endonuclease at the 5' end of the stretch of damaged DNA and Rad2 is a single strand endonuclease that cuts at the 3' end. RPA is thought to be the last player to arrive at the scene.

Many of these proteins also have roles in other cellular processes, such as recombination and transcription, therefore mutants express defects in several pathways. For a comprehensive review of NER, see Prakash and Prakash [17].

Most proteins participating in NER are present in *E. cuniculi*, with two exceptions. Half of a GGR heterodimeric damage sensor complex (Rad23) and the Tfb1 subunit of TFIIH appear to be absent (See Table 1). Rad23 appears to have diverse functions within the cell, ranging from DNA repair to the regulation of a cell-cycle checkpoint and protein degradation. Specifically, this protein helps to prevent the degradation of Rad4, as well as serving a role with the 26S proteasome in regulating the NER pathway [18,19]. Deletion of Tfb1 in *S. cerevisiae *is lethal [20], likely due to loss of function in transcription.

The presence or absence of Rad7 and Rad16 were not confirmed, as BLAST and PSI-BLAST searches using *S. cerevisiae *and *S. pombe *sequences as queries did not return homologues from most animals or other eukaryotes besides fungi.

### Methyltransferase Repair

Methyltransferases are present in both eukaryotes and prokaryotes and remove certain DNA lesions involving methylation (O^6^-methylguanine, O^4^-methylthymine). These proteins irreversibly relocate methyl groups from DNA to their own cysteine residues, and are therefore suicide enzymes [21].

*E. cuniculi *does not possess the methyltransferase found in other eukaryotes, Mgt1. Deletion of this gene is not lethal in *S. cerevisiae *[15].

### Mismatch repair (MMR)

In MMR, mismatches are recognized by the heterodimers MutSα (Msh2/Msh6) and MutSβ (Msh2/Msh3). Single base mismatches are recognized by MutSα and insertion/deletion loops (IDLs) less than about 9 nucleotides in length are recognized by MutSβ [22]. Both MutSα and MutSβ can recognize a single unpaired nucleotide. PCNA is also involved in MMR, perhaps assisting in these initial recognition steps. MutLα (Mlh1/Pms1) binds MutSα and β and allows them to efficiently bind to IDLs and mismatches. The exonuclease Exo1 then excises the mismatched base(s) and a DNA polymerase and DNA ligase fill and seal the gap.

It should be noted that the proteins required for the MMR process differ among eukaryotes. For instance, *Drosophila *and *Caenorhabditis *lack Msh3 homologues, and therefore do not require them for the removal of IDLs [22]. *Schizosaccharomyces *does possess a Msh3 homologue, but it appears to play a different role within the cell, instead participating in recombination [23].

The majority of *S. cerevisiae *MMR proteins are present in *E. cuniculi*. The sole missing protein is Msh3, a subunit of the MutSβ heterodimer that recognizes small IDLs (See Table 1). Deletion of this gene in *S. cerevisiae *is not lethal (See discussion) [20].

### Homologous recombination repair (HRR)

**Figure 2:**
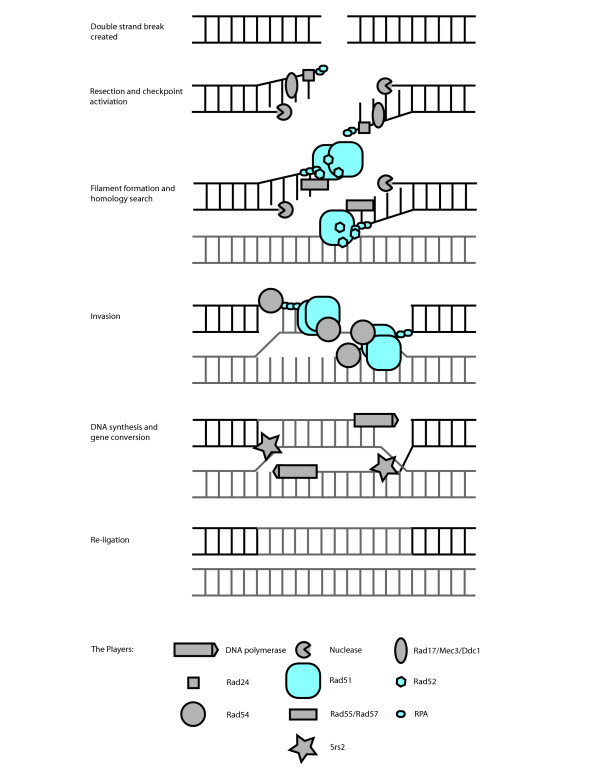
**The homologous recombination repair pathway**. (See text for explanation.) Blue proteins are present in *E. cuniculi*; all others are absent. Newly synthesized DNA is indicated in grey. Although the MRX complex (Mre11/Rad50/Xrs2) acts in damage recognition in this pathway, it is not shown. (Modified from Aylon and Kupiec [24].)

HRR is the major form of double strand break repair utilized in yeast. A double stranded break is recognized by damage recognition proteins, and single stranded overhangs are generated at both sides of the break. A region of the genome that is homologous to the single stranded overhangs is then found. Strand invasion follows, and the homologous (non-damaged) DNA is used as a template for synthesis on the broken strand. HRR is completed through re-annealing of the broken DNA strand and ligation. See figure 2 for an overview of this process.

The Rad51, 52, 54, 55 and 57 proteins perform most steps of the HRR process. Rad51 is a homologue of the bacterial enzyme RecA, and is well conserved within eukaryotes. When a double strand break is formed, the MRX complex (which is composed of Mre11, Rad50 and Xrs2, and also acts in NHEJ) is involved in damage recognition. The DNA ends on either side of the break are then chewed back in the 5' to 3' direction by an unknown nuclease. Rad24 (which is a checkpoint protein as well) is also involved in end processing. The results of this process are short 3' overhangs on either strand. RPA (which also acts in NER, as described above) then coats the overhangs. RPA is later replaced by Rad51, with the aid of Rad52, Rad55/Rad57, and very likely Rad54 as a genome-wide search for homologous sequences takes place. Strand invasion then occurs while the helicase Hpr5 removes Rad51 from the DNA. DNA is synthesized by an undetermined polymerase based on the donor template strands, and then ligated. Although the mechanism is not clear, it is evident that the Rad55/Rad57 complex is somehow involved in this last step. The Sgs1 helicase plays a specific role in the repair of double strand breaks generated by the stalling of a replication fork. For a review of HRR, see Aylon and Kupiec [24].

Three other signaling and damage sensor proteins are also involved in the HRR pathway, as well as the BER pathway and the NHEJ pathways. The Rad17/Med3/Ddc1 (9-1-1) complex triggers DNA damage checkpoints [25], and stimulates repair pathways [26] as well as various individual repair proteins, including DNA polymerase β [27], Rad51 [28] (HRR), Rad27 [29] (BER, NHEJ) and Cdc9 [30] (BER).

*E. cuniculi *lacks more than half of the proteins involved in the HRR pathway. Almost all steps of the process are affected by these losses (see discussion). Missing proteins include the Hpr5 helicase, Rad54 and Rdh54 (See Figure 2, Table 1). Rad24 and the 9-1-1 complex are all absent from the cell signaling pathways. *S. cerevisiae *single mutants lacking these proteins are viable [20], likely due to yeast's ability to use either double strand break repair pathway (HRR or NHEJ) to fix damaged DNA.

The presence or absence of Rad55 and Rad57 was not determined. Rad55 and Rad57 are paralogs of Rad51. PSI-BLAST searches using *S. cerevisiae *Rad55 and Rad57 proteins retrieve Rad51 in other fungi, therefore making it difficult to discern the presence of these proteins in *E. cuniculi*, which is related to fungi.

### Non-homologous end joining repair (NHEJ)

NHEJ is the second form of double strand break repair that is a separate, though not completely independent pathway from HRR. In *S. cerevisiae *this method of double strand break repair plays a minor role compared to the HRR pathway. Upon double strand break formation, damage is recognized and both ends of the lesion are brought together through the action of several proteins. A minimal amount of DNA synthesis occurs, which is followed by ligation. As DNA on either side of the break may be degenerated before the break is repaired, the potential for information loss in this case is substantial [24].

**Figure 3:**
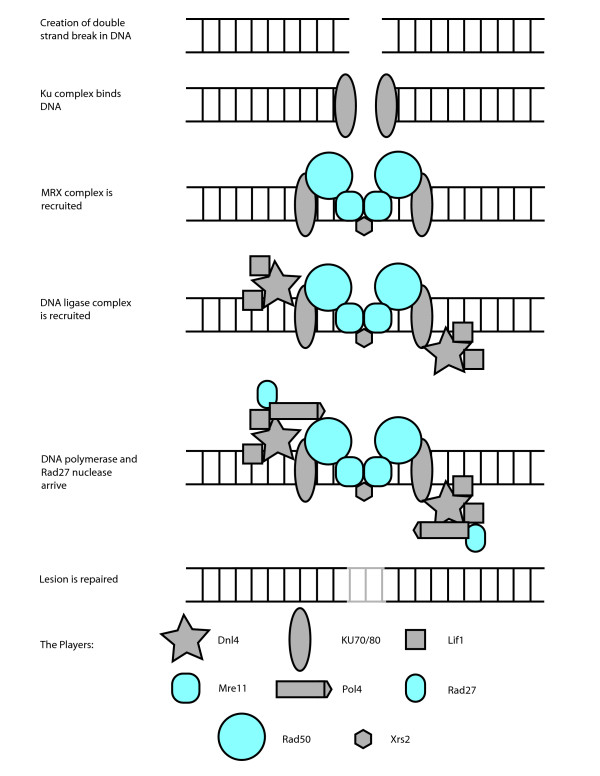
**The non-homologous end joining repair pathway**. (See text for explanation.) Blue proteins are present in *E. cuniculi*; all others are absent. Newly synthesized DNA is indicated in grey. (Modified from Hefferin and Tomkinson [32].)

The NHEJ process begins when the Ku complex (Ku70/Ku80) binds either end of the double strand break (See Fig 3). These proteins are DNA-dependent protein kinases that also have a role in telomere maintenance. Once bound to the damaged site, the Ku complex is responsible for recruiting the MRX complex for the next stage in the repair process. The MRX complex is composed of Rad50 (an ATP binding protein), Mre11 (a 5'-3' exonuclease) and Xrs2 (responsible for aligning the MRX complex with the break site) [31]. Dnl4/Lif1 (a DNA ligase complex) is tethered to the break site by Xrs2 and the Ku complex. The DNA polymerase Pol4 and the structure-specific nuclease Rad27 are the last players to arrive at the scene, thus completing the repair complex.

All of the yeast NHEJ proteins are present in most eukaryotes, and the core of Ku70 and 80 is homologous to a smaller bacterial protein that performs the same function, thus indicating a large degree of conservation. For a review of this process, see Hefferin and Tomkinson [32].

*E. cuniculi *is missing nearly all NHEJ proteins. Absent proteins include Ku70, Ku80, Xrs2, Dnl4, and Pol4 (See Fig 3, Table 1). As is the case with single *S. cerevisiae *mutants for genes involved in the HRR pathway, most are viable [20] due to yeast's ability to rely on the other (HRR) double strand break repair pathway.

Although there are animal homologues of Lif1 and Xrs2 (Xrcc4 and Nbs1, respectively), BLASTP and PSI-BLAST searches using yeast proteins did not retrieve homologues in any organisms other than fungi. The presence or absence of these proteins is therefore not known.

### DNA polymerases

DNA polymerases are essential for both genome replication and repair. There are several polymerases present in eukaryotic cells, all of which serve particular functions within the cell. The polymerases α, δ and ε act in the process of genome replication, but also play roles in certain repair processes, notably NER and HRR. Polymerase γ acts solely within mitochondria, while all other polymerases are nuclear. Polymerase β is a specialized repair polymerase that is involved in BER and NHEJ. The polymerases ζ, η and Rev1 help prevent double stranded DNA breaks from forming during replication due to their ability to synthesize DNA through a lesion, where polymerases α, δ and ε stall and dissociate from the replication fork [4].

Of the 8 polymerases identified in *S. cerevisiae *that have human counterparts (confirming that they are not fungal or ascomycete specific), *E. cuniculi *possesses 3: α, δ and ε (See Table 1). All three of these polymerases are necessary for viability in *S. cerevisiae*. All of the polymerases that are absent in *E. cuniculi *are utilized solely for repair or lesion bypass and are not essential for viability, likely because their function is replaced by other polymerases [20].

## Discussion

In general, the consequences of genome reduction on DNA repair in *E. cuniculi *are most evident in the double strand break pathways. The single strand repair pathways have been less affected, but some are operating at a level of reduced complexity compared to *S. cerevisiae*.

### Reduction in complexity of DNA repair

*E. cuniculi*'s BER pathway lacks the DNA ligase Cdc9, DNA polymerase β and Mms4. Although deletion of Cdc9 is lethal in *S. cerevisiae *[20], the role of this protein is likely filled by another ligase. This is not unusual in *S. cerevisiae*, as several enzymes are sometimes able to act on the same substrate. For example, in the HRR pathway, the polymerase and nuclease have not yet been defined, likely because different combinations of polymerases and nucleases are capable of performing the required functions of this pathway [24]. The absence of DNA polymerase β could indicate that most BER in *E. cuniculi *is carried out via the long patch pathway, where DNA is synthesized by the polymerases δ and ε. The use of one BER pathway over another is common in eukaryotes; studies have indicated that in yeast, long patch BER is carried out preferentially instead of short patch BER, whereas in humans, the reverse is true [13]. The absence of Mms4 is not likely to have serious ramifications for BER in *E. cuniculi*. The Mus81-Mms4 endonuclease processes 3' ends of nicked DNA to prepare for DNA synthesis. However, its role is predicted to be minor, and somewhat overlapping with that of the Rad1-Rad10 endonuclease, which is present [12].

The NER pathway is missing a core TFIIH component and the Rad23 subunit of the Rad4/Rad23 damage recognition complex. TFIIH is composed of a ring containing the three Tfb proteins (Tfb1, Tfb2, and Tfb3), which serve to tether the functional parts of the complex: the helicases Rad3 and Rad25 [33]. Since transcription must occur in *E. cuniculi*, it is difficult to predict exactly how the absence of these proteins would affect this organism, as deletion of Tfb1 is lethal in *S. cerevisiae*. Complete absence of this protein is difficult to reconcile with the Tfb ring's essential functions, as well as the presence of the two other ring components (See below). However, it is not unreasonable to assume that the absence of Tfb1 would likely lead to a reduction in the efficiency of this repair process, particularly when Rad23 also appears to be absent.

*E. cuniculi *also lacks Msh3, which interacts with Msh2 to form MutSβ, which recognizes insertion or deletion loops (IDLs) in the MMR pathway. In *S. cerevisiae*, deletion of Msh3 is not lethal, but mutants are slightly more prone to frameshift mutations [15]. Although the MutSβ heterodimer is present in *S. cerevisiae*, *Schizosaccharomyces*, humans and *Arabidopsis*, its presence is not ubiquitous among eukaryotes. *Drosophila *and *Caenorhabditis *lack Msh3, where it appears that the MutSα complex is able to recognize both mismatches and insertion or deletion loops [22].

*Drosophila *and *Caenorhabditis *are able to effectively perform MMR in the absence of Msh3, which is the sole missing protein in *E. cuniculi*. Therefore, it is very likely that this pathway operates in *E. cuniculi *in a similar manner to these organisms, whose MMR systems are fully functional.

The absence of the DNA methyltransferase Mgt1 suggests that *E. cuniculi *is able to employ other methods to remove O^6^-methylguanine from its DNA. In the bacterium *Escherichia coli*, O^6^-methylguanine can be removed by both the NER and the methyltransferase mechanisms [34], therefore it is likely that *E. cuniculi *has simply dispensed with one of two parallel pathways.

### DNA polymerases and repair

Eukaryotes and prokaryotes possess many specialized DNA polymerases to accomplish specific tasks within the cell. Some of these polymerases are involved in genome replication, while others act solely in repair processes.

*E. cuniculi *possesses only three DNA polymerases (α, δ and ε) of the 8 present in *S. cerevisiae*. All of these polymerases are involved in standard genome replication, while polymerase δ also plays a role in BER, NER, MMR and in bypassing DNA lesions [4]. Polymerase ε is required for BER and probably NER. Polymerases α, δ and ε are all likely utilized in HRR [4]. *E. cuniculi *lacks polymerase β, which is utilized in a variety of repair pathways and polymerases ζ and η, which are used for error-prone and error-free DNA synthesis across lesions, respectively [4]. *E. cuniculi *also lacks the mitochondrial DNA polymerase γ.

*S. cerevisiae *mutants lacking polymerase β display a high frequency of recombination and sensitivity to methyl methanesulfonate (MMS). Rev1 mutants display decreased revertibility, while polymerase η mutants have a heightened sensitivity to UV radiation. Conversely, polymerase ζ deletion mutants resist UV mutagenesis. Cells lacking polymerase γ lose their mitochondrial DNA [15], however microsporidian mitochondria (mitosomes) are highly reduced and it is unlikely that they possess autonomous DNA [35]. The phenotype of a *S. cerevisiae *cell lacking several polymerases is not known, but one could speculate that such cells would display a higher frequency of double stranded DNA breaks generated during replication due to a lack of translesion polymerases.

### Double strand break repair in *E. cuniculi*

The fact that most of the NHEJ repair proteins appear to be absent in *E. cuniculi *is perhaps not overly surprising, as this method of double strand break repair appears to be a back-up method in yeast [24]. (Note that this preference is not strictly maintained throughout eukaryotic life. Humans, for example, use NHEJ as the primary pathway [1].) *E. cuniculi*'s genome is known to be highly reduced compared to that of *S. cerevisiae*. Therefore, it seems logical that the first genes to be deleted from a genome undergoing reduction would be those encoding proteins that act in back-up pathways.

Of key interest is the lack of Ku proteins (Ku70 and Ku80) in *E. cuniculi*. These proteins play a pivotal role in NHEJ; they are involved in recognizing double strand break sites and in recruiting other repair factors to the break site. Not only is their function key, but they are present in archaebacteria, bacteria and eukaryotes. The core of the Ku proteins is largely conserved from prokaryotes to eukaryotes [32]. However, the absence of these proteins in *E. cuniculi *is not entirely unique, as we were also unable to identify Ku proteins in the genome of the human parasite *Plasmodium*, nor has it been recognized in *Trichomonas *[36].

Dispensing with a backup double strand break repair pathway during genome reduction would stand to reason if the primary repair pathway was retained, however, this is also highly questionable. *E. cuniculi *also lacks over half of the HRR proteins that are present in yeast (See Table 1).

The DNA helicase Hpr5, Rad54, Rdh54 and the checkpoint/DNA end-processing Rad24 are among the proteins that appear to be absent from the HRR pathway. Hpr5 plays a cryptic role in HRR, as *S. cerevisiae *deletion mutants have hyperrecombination phenotypes [37], and the protein was therefore assumed to be a negative regulator of the process. However, recent work by Aylon et al. [38] has shown that Hpr5 is intimately involved in commitment to gene conversion, which must take place before recombination can occur. Rdh54 is a Rad54 homolog that participates in interhomologue gene conversion and meiosis [39], while Rad54 is a chromatin remodeling protein that has been implicated in strand invasion and the removal of repair proteins from DNA after HRR has taken place [40]. In addition to functioning as a checkpoint protein, Rad24 also plays a role in the resection and recombination processes [41].

It is possible that the functions of Rad55 and Rad57 (which are potentially absent) are carried out by Rad51, as all three proteins are homologues of the bacterial protein RecA. This is a distinct possibility, as Rad55 and Rad57 appear to act in concert with Rad51 during the HRR process [24].

Although the Rad51, Rad52 and Sgs1 proteins are present in *E. cuniculi*, it is not known whether HRR can take place in the absence of all other HRR components. It is difficult to imagine this process occurring in the absence of DNA resection (Rad24), strand invasion (Rad54) and gene conversion (Hpr5 and Rdh54).

Therefore, *E. cuniculi *appears to have drastically reduced both mechanisms for double strand break repair. Although *E. cuniculi*'s genome contains very few duplicate genes (regions of homologous sequence) to use as templates for DNA synthesis in HRR, both *S. cerevisiae *[42] and mammals [43] prefer to use sister chromatids rather than homologous sequences (on the same or different chromosomes) for this process. As *E. cuniculi *is likely diploid [44] (as are yeast and mammals), it is reasonable to assume that this preference would exist in this organism as well.

Given that such a large number of genes involved in both double strand break repair pathways are absent, it is curious that some of these genes have been retained. When one looks closely at the functions of these genes, it is evident that they all play roles in other critical biological processes. Mre11 and Rad50, both members of the MRX complex (found in both double strand break repair pathways), are also involved in telomere maintenance and the generation of meiotic double strand breaks [45,46]. Rad27 is a nuclease that is implicated in the processing of Okazaki fragments during replication [47].

All of the proteins belonging to the HRR pathway that are present in *E. cuniculi *are also involved in meiosis [45]. Although sexual reproduction has not been observed in *E. cuniculi*, it does contain three of the seven core meiosis-specific genes (*Hop2*, *Mnd1 *and *Spo11*), as discussed in Ramesh et al. [48], and there is evidence that it may possess a mating type locus [49]. Sexual reproduction has also been observed in numerous other microsporidia [5], therefore there is little reason to suspect that *E. cuniculi *is an exception. As a large number of proteins involved in the HRR pathway are absent in *E. cuniculi*, the repair functions of the remaining proteins are unknown. It is possible that they have been retained because of their role in meiosis.

### Potential consequences for *E. cuniculi*

Reductions within the DNA repair pathways have led to two fundamentally different outcomes: reduced complexity by loss of a few proteins (NER, MMR, BER) and drastic losses of half or more proteins involved in a pathway (methyltransferase repair, HRR and NHEJ). Although an organism may be able to tolerate a somewhat sloppy repair system, it is difficult to imagine how the organism could exist without any means to mend double-strand DNA breaks, especially given their frequency during meiosis and mitosis. *E. cuniculi *must, therefore, utilize some other form of double strand break repair, or contain such highly divergent copies of most NHEJ and HRR proteins that they were impossible to identify in this study.

Along with many of the proteins that carry out the work of repair, *E. cuniculi *has lost several proteins that participate in cell signaling and cycle control. The 9-1-1 signaling complex is absent, which has been proposed to play a role in the signaling cascade leading to cell cycle arrest and apoptosis [50]. Both the Ku and MRX complexes are also involved in cell cycle control, although their roles are not well defined [51]. Loss of coordination of cellular activities could result from the absence of these proteins.

In addition to their role in repair, the Ku proteins protect telomeres from degradation and help to control telomerase activity [52]. As *E. cuniculi *houses eleven chromosomes that contain telomeres [44], and encodes the catalytic subunit of the telomerase enzyme [6], this organism must have developed an alternate method to maintain its telomeres, or it would suffer extreme telomere attrition.

Like the Ku proteins, the DNA ligase Cdc9 performs several functions as well. It plays a role in recombination and in the ligation of Okazaki fragments during replication [53], therefore, it is possible that these processes are somewhat impaired.

The reduction that is observed within the DNA repair pathways is similar to that observed throughout *E. cuniculi*'s genome, as this organism lacks many proteins that participate in diverse biosynthetic pathways. In this way, the genome of *E. cuniculi *is very similar to those of many endosymbiotic and parasitic bacteria. *Buchnera aphidicola *has also lost many DNA repair genes during the process of genome reduction; indeed it has been proposed that it was this lack of DNA repair genes that allowed *Buchnera*'s genome to become so small in the first place [54].

We cannot rule out the possibility that our bioinformatics tools were unsuccessful in locating highly divergent proteins that act in the DNA repair processes. It is also possible that in some cases, other non-homologous proteins carry out essential functions to replace absent proteins (ie. Tfb1 in NER). Such proteins may still be identified, as roughly half of *E. cuniculi*'s genome consists of hypothetical proteins [6]. Another potential explanation for this lack of biosynthetic machinery is that *E. cuniculi *is able to import many of the products of these pathways from the host's cytoplasm (ie., ATP) [5]. However, it seems unlikely that this would be the case for DNA repair proteins, as protein uptake has not been documented in microsporidia, and these proteins would have to be targeted to the nucleus in order for them to function. For the moment, double strand break repair in *E. cuniculi *will remain a mystery.

## Conclusion

Our survey of *E. cuniculi*'s DNA repair genes indicates that the process of genome reduction has affected all major DNA repair pathways. All of the single strand repair pathways (BER, NER and MMR) have lost at least one component, indicating that these pathways are less complex than in *S. cerevisiae*, and could be less efficient. All replicative DNA polymerases are present in *E. cuniculi*, although the specialized repair polymerases are absent. The absence of these enzymes could lead to inefficient DNA damage repair and creation of double stranded DNA breaks that are not easily repaired. Surprisingly, more than half of the proteins participating in both double strand break repair pathways (HRR and NHEJ) and the sole component involved in methyltransferase repair are absent in *E. cuniculi*. The proteins that remain are all involved in additional cellular functions (such as meiosis).

## Methods

### Identification of DNA repair pathway components in *S. cerevisiae *and data mining in *E. cuniculi*

Components of the six major DNA repair pathways were gathered from recent literature and supplemented with data from the *Saccharomyces *genome database [15]. Refer to Table 1 for a list of genes involved in each pathway and the DNA polymerase subunits and references.

Amino acid sequences of DNA repair proteins from *S. cerevisiae *(Table 1) were collected from NCBI GENBANK, and compared to *E. cuniculi*'s protein and nucleotide data using BLASTP and TBLASTN [55]. In instances where a *Schizosaccharomyces pombe *homologue to an *S. cerevisiae *protein existed that was more conserved among eukaryotes than the *S. cerevisiae *protein itself, the *S. pombe *sequence was used in the BLAST searches. (These proteins are indicated with asterixes in Table 1 and *S. pombe *protein ID numbers are given).

In most instances, BLASTP searches were sufficient to identify putative *E. cuniculi *homologues. In cases where no significant results (significance was defined arbitrarily as an e-value of 10^-5 ^or less) were produced from the initial BLASTP analysis, the PSI-BLAST algorithm was used. Homologues of the *S. cerevisiae *protein were identified in all available eukaryotic protein data to construct a position-specific scoring matrix [55]. Up to six iterations were run in cases where no significant *E. cuniculi *alignment was found. In order to rule out similarity by chance, the identities of putative homologues detected in *E. cuniculi *were confirmed by comparing them to GENBANK's *S. cerevisiae *protein database using BLASTP. Homology was inferred when this search recovered the *S. cerevisiae *protein that was used for the initial *E. cuniculi *search as the top hit.

In many instances, BLAST searches in *E. cuniculi *confirmed annotations of DNA repair genes and polymerases by Katinka et al. [6].

A brief examination of the number of interaction partners of each protein in *S. cerevisiae *was conducted using data from the online Database of Interacting Proteins (DIP) [56]. Proteins that are absent in *E. cuniculi *do not have a significantly different number of interaction partners from proteins that are present. (Data not shown.)

## Authors' contributions

EEG conceived of the study, performed data mining and analyses and drafted the manuscript. NMF assisted in analyses and made critical contributions to the manuscript. Both authors read and approved the final manuscript.

## Figures and Tables

**Table 1:** *S. cerevisiae *DNA polymerases and proteins that participate in the five primary DNA repair pathways.

**Base Excision Repair (BER)**				**Nucleotide Excision Repair (NER)**			
	
**Gene Name**	**Present in *E. cuniculi***	***S. cerevisiae *Protein ID**	***E. cuniculi *Protein ID**	**Gene Name**	**Present in *E. cuniculi***	***S. cerevisiae *Protein ID**	***E. cuniculi *Protein ID**
							
	
Apn1	Y	CAA81954	CAD26065	Rad1 (XPF)	Y	P06777	CAD26381
Apn2	Y	NP_009534	NP_585892	Rad2 (XPG)	Y	CAA97287	CAD27314/XP_955715
Mag1	Y	NP_011069	CAD25924/CAD26679	Rad3 (Ercc2/XPD)	Y	CAA46255	NP_585776
Mus81	Y	NP_010674	NP_584664	Rad4 (XPC)	Y	CAA39375	CAD24914
Ntg1	Y	NP_009387	CAD26394	Rad10 (Ercc1)	Y	CAA86642	CAD25852/NP_586248
Ogg1	Y	NP_013651	CAD26383	Rad14 (XPA)	Y	P28519	NP_586232
PCNA	Y	AAS56041	NP_597446	Rad25 (Ssl2/XPB)	Y	Q00578	CAD24977
Rad1 (XPF)	Y	P06777	CAD26381	Rad26 (Ercc6/CSB)	Y	CAA57290	CAD27013/XP_955594
Rad10 (Ercc1)	Y	CAA86642	CAD25852/NP_586248	Rpa1	Y	NP_009404	CAD25779
Rad27 (Fen1)	Y	CAA81953	CAD26252	Rpa2	Y	NP_014087	CAD25396
Ung1	Y	CAA86634	CAD26772	Ssl1	Y	CAA97527	CAD25215
*Xrcc1 (Cut5)	Y	P32372	NP_584657	Tfb2	Y	AAB40628	CAD24937
**Cdc9 (DNA lig I)**	**N**	**CAA48158**	**-**	Tfb3	Y	AAB64899	CAD25932
**Ddc1 (Rad9)**	**N**	**NP_015130**	**-**	Tfb4	Y	NP_015381	CAD25620
**Mec3 (Hus1)**	**N**	**NP_013391**	**-**	Rad7	?	*CAA85071*	*-*
**Mms4**	**N**	**NP_009656**	**-**	Rad16	?	*CAA89580*	*-*
**Rad17 (Rad1)**	**N**	**CAA99699**	**-**	**Rad23 (HR23B)**	**N**	**AAB28441**	**-**
				**Tfb1**	**N**	**AAB64747**	**-**
	
**Methyltransferase Repair**				**DNA Polymerases**			
	
**Gene Name**	**Present in *E. cuniculi***	***S. cerevisiae *Protein ID**	***E. cuniculi *Protein ID**	**Gene Name**	**Present in *E. cuniculi***	***S. cerevisiae *Protein ID**	***E. cuniculi *Protein ID**
	
**Mgt1**	**N**	**CAA42920**	**-**	α Pol1	Y	AAZ22505	CAD26619
				
				α Pol12	Y	NP_009518	CAD25827
				
**Mismatch Repair (MMR)**				α Pri1	Y	AAT92878	CAD26368
				
**Gene Name**	**Present in *E. cuniculi***	***S. cerevisiae *Protein ID**	***E. cuniculi *Protein ID**	α Pri2	Y	NP_012879	CAD26641
				
				δ Pol3	Y	CAA43922	CAD27015
Exo1	Y	NP_014676	CAD25986	δ Hys2	Y	NP_012539	CAD27050
Mlh1	Y	NP_013890	NP_597370	ε Dpb2	Y	NP_015501	NP_597373
Msh2	Y	CAA99102	CAD26200/NP_597565	ε Pol2	Y	NP_014137	CAD25840
Msh6	Y	NP_010382	NP_586186	**β Pol4**	**N**	**NP_009940**	**-**
PCNA	Y	AAS56041	NP_597446	**γ Mip1**	**N**	**NP_014975**	**-**
Pms1	Y	P14242	NP_586432	**η Rad30**	**N**	**NP_010707**	**-**
**Msh3**	**N**	**CAA42247**	**-**	**ζ Rev3**	**N**	**NP_015158**	**-**
				**ζ Rev7**	**N**	**AAA98667**	**-**
				**Rev1**	**N**	**CAA99674**	**-**
	
**Homologous Recombination (HRR)**				**Non-Homologous End Joining (NHEJ)**			
	
**Gene Name**	**Present in *E. cuniculi***	***S. cerevisiae *Protein ID**	***E. cuniculi *Protein ID**	**Gene Name**	**Present in *E. cuniculi***	***S. cerevisiae *Protein ID**	***E. cuniculi *Protein ID**
	
Mre11 (Rad32)	Y	BAA02017	CAD26648/NP_597471	Mre11 (Rad32)	Y	BAA02017	CAD26648/NP_597471
Rad50	Y	CAA65494	CAD25593/NP_585989	Rad27 (Fen1)	Y	CAA81953	CAD26252
Rad51	Y	CAA45563	CAD25992/NP_586388	Rad50	Y	CAA65494	CAD25593/NP_585989
Rad52	Y	CAA86623	XP_955647	Lif1 (Xrcc4)	?	*NP_011425*	*-*
Rpa1	Y	NP_009404	CAD25779	*Xrs2 (Nbs1)	?	*BAC80248*	*-*
Rpa2	Y	NP_014087	CAD25396	**Ddc1 (Rad9)**	**N**	**NP_015130**	**-**
Sgs1	Y	NP_013915	CAD25646	**Dnl4**	**N**	**CAA99193**	**-**
Rad55 (Rad51C)	?	*BAA01284*	*-*	**Mec3 (Hus1)**	**N**	**NP_013391**	**-**
Rad57 (Xrcc3)	?	*NP_010287*	*-*	**Ku70**	**N**	**NP_014011**	**-**
*Xrs2 (Nbs1)	?	*BAC80248*	*-*	**Ku80**	**N**	**NP_013824**	**-**
**Ddc1 (Rad9)**	**N**	**NP_015130**	**-**	**Rad17 (Rad1)**	**N**	**CAA99699**	**-**
**Hpr5 (Srs2)**	**N**	**CAA89385**	**-**				
**Mec3 (Hus1)**	**N**	**NP_013391**	**-**				
**Rad17 (Rad1)**	**N**	**CAA99699**	**-**				
**Rad24 (Rad17)**	**N**	**P32641**	**-**				
**Rad54**	**N**	**CAA88534**	**-**				
**Rdh54 (Rad54B)**	**N**	**CAA85017**	**-**				

Presence or absence in *E. cuniculi *is indicated (as defined in Methods), along with the genbank accession numbers for both *S. cerevisiae *and *E. cuniculi *proteins. Absent proteins are presented in bold type. Italicized accession numbers indicate that the presence or absence of these proteins in *E. cuniculi *was unclear. (See results for more information.) When *S. pombe *proteins appeared to be more conserved among eukaryotes than *S. cerevisiae *homologues (or where *S. cerevisiae *homologues do not exist), they were used to conduct the BLAST and PSI-BLAST searches. These proteins are marked with asterixes. *S. cerevisiae *nomenclature is used, with *S. pombe *or animal homologues given in brackets. Pathway components were largely compiled from the following sources: MMR from Marti et al. [22], BER from Boiteux and Guillet [12], NER from Prakash and Prakash [17], NHEJ from Daley et al. [57], HRR from Aylon and Kupiec [24], DNA polymerases from Burgers [58] and Hubscher et al., [4].
